# Association of American Medical Editors

**Published:** 1874-05-01

**Authors:** 


ASSOCIATION OF AMERICAN MEDICAL EDITORS.—The next annual meeting of this Society is to be held in Detroit, Monday evening, June 1st, 1874, the evening before the commencement of the session of the American Medical Association. The objects for which the Association of Medical Editors was formed were good, and of sufficient importance to justify the organization. But thus far the number of those connected with the medical press who have attended the meetings, has been so limited as to defeat altogether the most important of these objects. We hope a larger attendance will be present, in Detroit. If not, it is questionable whether the Association should be perpetuated.

